# Minimum meal frequency practice and associated factors among children aged 6–23 months old in The Gambia: a multilevel mixed effect analysis

**DOI:** 10.1038/s41598-023-49748-0

**Published:** 2023-12-19

**Authors:** Bewuketu Terefe, Mahlet Moges Jembere, Birhanu Abie Mekonnen

**Affiliations:** 1 Department of Community Health Nursing, School of Nursing, College of Medicine and Health Sciences, University of Gondar, P.O. Box: 196, Gondar, Amhara Ethiopia; 2 Department of Emergency and Critical Care Nursing, School of Nursing, College of Medicine and Health Sciences, University of Gondar, Gondar, Amhara Ethiopia; 3 Department of Pediatrics and Child Health, School of Medicine, College of Medicine and Health Sciences, University of Gondar, Gondar, Amhara Ethiopia

**Keywords:** Biomarkers, Medical research

## Abstract

A proxy measure for a child's energy needs, minimum meal frequency (MMF) looks at how often children were fed things other than breast milk. Infants and young children who do not consume enough food frequently are more likely to suffer from malnutrition, which can lead to increased morbidity and mortality as well as stunting and micronutrient deficiencies. There is no MMF recommended by studies in The Gambia. Hence, the purpose of this study was to evaluate the practice of MMF and the factors that influence it in children aged 6–23 months in The Gambia. Data from The Gambian Demographic and Health Survey (GDHS-2019/20) were used to identify factors affecting the MMF at individual and community levels. A multi-level regression model and weighted samples of 2100 children were employed for the investigation. After being examined by a *p*-value of < 0.25 in the binary regression, factors with a *p*-value of < 0.05 were judged statistically significant. This study found that about 57.95% had provided MMF. Primary and secondary educated mothers (aOR = 1.44, CI 1.11, 1.87), and (aOR = 1.43, CI 1.09, 1.86), wealthiest (aOR = 1.76, CI 1.04, 2.99), 35–49 years old mothers (aOR = 1.35, CI 1.01, 1.79), female household head (aOR = 0.72, CI 0.53, 0.98), breastfeeding status(aOR = 0.10, CI 0.07, 0.15), currently working (aOR = 1.27, CI 1.04, 1.56), 12–17 months child (aOR = 1.40, CI 1.13, 1.73), 18–23 months child (aOR = 1.44, CI 1.08, 1.91) have shown association with MMF. Regarding regions Mansakonko, Kerewan, Kuntaur, and Janjanbureh local government areas have shown (aOR = 3.51, CI 1.77, 6.97), (aOR = 5.17, CI 2.67, 9.99), (aOR = 2.26, CI 1.14, 4.47), and (aOR = 2.35, CI 1.19, 4.64) as compared to Banjul local government area. Comparing MMF in The Gambia to WHO standards, it must be considered low. Encouragement of women and coordinated enhancement of the current nutritional intervention are therefore effective in boosting children's consumption of a variety of foods.

## Introduction

Appropriate feeding practices for infants and young children can increase child survival and promote healthy growth and development, particularly during the critical period from birth to age two. With appropriate minimum meal frequency (MMF) and continuous breastfeeding and the consumption of appropriate, sufficient, and safe supplemental foods, children's health and growth are improved as they reach the age of six months, perhaps reducing stunting throughout the first two years of life^1^. Beginning at six months, breastfeeding should be combined with age-appropriate, safe solid, semi-solid, and soft food feeding of MMF. Specific guidelines are available about how the feeding should be done, covering everything from food consistency to responsive feeding^2–4^.

The MMF study counts the number of times children received foods other than breast milk as a measure of their energy needs. The minimal amount needed depends on the child's age and whether it is breastfed. If breastfed children get solid, semisolid, or soft foods at least twice daily for infants aged 6–8 months and at least three times daily for kids aged 9–23 months, they are regarded to be receiving the recommended MMF. If non-breastfed children aged 6–23 months get solid, semisolid, or soft foods at least four times daily, they are deemed to be fed with a MMF^5,6^.

Around 35% of child fatalities under the age of five globally are directly or indirectly attributable to undernutrition. In low- and middle-income nations, maternal and child undernutrition are extremely common, which contributes to significant increases in childhood impairment, mortality and the global burden of disease^5,7^. While a World Health Organization report1 revealed that 10% (or 55 million) of children under the age of five in poor nations were wasted and 32% (186 million) of children under the age of five were stunted^5^. In developing nations, malnutrition is a significant issue with regard to community health. Malnutrition would result from a combination of inadequate child feeding practices and household food instability^8^.

According to the UNICEF MICS report from 2018, malnutrition continues to be a major issue for The Gambian children. Most children did not receive adequate nutrition in the form of vitamins, proteins, and minerals due to poor health care and parents' lack awareness^8^. So that stunting and underweight are the headache of The Gambian children with a prevalence of 18% and 12% respectively^9,10^. This finding is corroborated by the 2021 Global Nutrition Report, which noted that The Gambia has not made much progress in reducing non-communicable diseases linked to diet and under-five stunting^11^. The country's economy is characterized by traditional subsistence agriculture, with a historic reliance on groundnuts (peanuts) for export earnings. The population is predominantly rural, with over 63% living in rural villages^12–14^. The Gambian economy is heavily dependent on peanut production and export. The country also relies on fishing and tourism. The main agricultural products include peanuts, millet, sorghum, rice, corn, and cassava. The country's natural resources include fish, clay, silica sand, titanium, tin, and zircon. The land use is dominated by agricultural land, with a significant portion dedicated to arable land and permanent pasture^12–14^. Regarding education, the overall literacy rate in The Gambia is around 50%, with a significantly lower rate for women than for men. Education is theoretically free and universal for primary school, but in practice, there are disparities between urban and rural areas. The literacy rate is 38.6% for the total population, with 52.8% for males and 24.9% for females^13–15^.

Numerous researches have shown that sociodemographic factors and meal frequency are related. Low levels of parental education, mother's age^9,16,17^, mother's work^18^, children's age and birth order^16,17^, household wealth status^9,16^, family size^16^, and place of residence^17^ were thus substantially linked to low levels of dietary diversity practices. Additionally, the minimal meal frequency was substantially correlated with the birth interval^18,19^, satisfactory media exposure^16,17^, mother's participation in decision-making^17^, and maternal antenatal (ANC) and postnatal (PNC) care follow ups^19,20^. Undernutrition is still a big concern for the general population and is regarded as a key measure of a nation's progress. In order for this study to be important in offering the necessary data by considering individual community level factors that might affect MMF. This is so that the government, stakeholders, policy makers, nutritionists, and healthcare professionals can be encouraged to concentrate on the nutritional condition of the children by having evidence of MMF and associated variables among children in The Gambia aged 6–23 months. Based on this evidence, they might also come up with various plans to stop the morbidity and death linked to the children's improper feeding practices.

## Methods and materials

### Study design and setting

This study was built on The Gambia Demographic Health Survey (GDHS), a secondary, substantial community-based cross-sectional survey that was conducted in The Gambia from November 21, 2019, to March 30, 2020. Between October 2019 and February 2020, The Gambia Bureau of Statistics (GBoS) conducted the 2019–2020 The Gambia Demographic and Health Survey (GDHS 2019–2020) in partnership with the Ministry of Health and Social Welfare. The second DHS survey to be carried out in The Gambia in collaboration with the global Demographic and Health Survey Program is known as The Gambia Demographic and Health Survey (GDHS 2019–2020). A nation in West Africa is called The Gambia. The Gambia is a small West African country situated on the Atlantic coast, surrounded by Senegal. It is Africa's smallest non-island country and one of the most densely populated. The major ethnic groups include Mandinka, Wolof, Fulani, Diola, and Soninke peoples. The population is predominantly Muslim, with a small Christian minority. The Atlantic Ocean serves as its Western border, while the Republic of Senegal constitutes its northern, southern, and eastern borders. The two distinct seasons in the country are the dry season (November–May) and the rainy season (June to October)^10^.

### Source and study population

Every eligible mother who has a child in The Gambia, aged 6–23 months, in the selected clusters was considered to be a study population. The source population consisted of women who had children aged 6–23 months in the 24 h before to the survey^6^. In order to account for any unequal sample distributions during data collection, weightings for the children's sample were used throughout the estimation process. Consequently, 2100 weighted samples of children between the ages of 6 and 23 months were used in the study.

### Sample size determination, and sampling methods

Stratified, two-stage cluster sampling was used for the survey. Enumeration areas (EAs) for each sampling stratum were initially chosen with a probability proportional to their size. In the first stage, a probability corresponding to each cluster's size within each sampling stratum was used to choose the EAs. 281 EAs in all were selected. From the resulting lists of households, a predetermined number of twenty-five were systematically selected to serve as the sampling frame. This resulted in a total sample size of seventy-five selected households. The findings from this sample are representative of findings from local government units, rural areas, and cities across the country^10^. The children's sample weightings were added during the estimation procedure to account for differential sample distributions during data collecting. Thus, 2100 weighted samples of children between the ages of 6 and 23 months were included in the research. The GDHS report has access to the information. In the second stage, each household was meticulously sampled. On each of the chosen clusters, an operation to list the households was run. Results from this sample are typical of findings in local government and metropolitan settings across the nation. More information is available from GDHS reports^6,10^.

### Data collection tool, quality control, and procedures

Before any data was collected, a pre-test was carried out in the DHS, field workers who had participated in the pre-test were debriefed, and any necessary modifications to the questionnaires were made. For further information on the data gathering procedure, see the DHS guidelines^6^. Approximately every five years, the DHS uses five different surveys to gather data from professionally trained data collectors that are nationally representative and reflect the distinct health and demographic issues that each country faces. These comprised biomarkers, health facilities, and questionnaires for men, women, and households. The kids record questionnaire was utilized in this study to determine the consumption of MMF in The Gambia, taking into account contributing factors at the individual and community levels for young children aged 6–23 months^6,21^.

### Study tools and measurement

#### Outcome variable

Minimum Meal Frequency (MMF). The minimum is defined as: twice for breastfed infants aged 6–8 months, three times for breastfed children aged 9–23 months, and four times for non-breastfed infants aged 6–23 months for the proportion of children aged 6–23 months who receive solid, semisolid, or soft foods (but also including milk feeds for non-breastfed children)^6,22^.

#### Independent variables

A review of the literature on the factors connected to children's MMF served as the foundation for the study's design and conceptual framework. In order to determine the most likely causes of MMF in The Gambia, features at the individual and community levels were assessed. Among the factors at the individual level were maternal age (15–24, 25–34, and 35–49 years old), educational attainment (not educated, primary, secondary, and higher level), the sex of the household head (male, female), wealth index (poorest, poorer, middle, richer, and richest), marital status, (not married, married) use of contraception, (no, yes), child age in months (6–11, 12–17, and 18–23), ANC visit (less than four, and greater than four), PNC after two months (no, yes), place of birth (home, health facility), family size (2–6, 7–10 and > 10), twins (no, yes), under-five children (< 3, and more than three), child size at birth (small, average, and large), watching to Television (no, yes), listening to radio (no, yes), reading to magazine/newspaper reading (no, yes), sex of the child (male, female), breastfeeding status (no, yes), order of birth (first, 2nd or 3rd, and 4th or above orders), currently working status (no, yes), and use of contraception (no, yes). On the other hand, characteristics associated to the community level include the use of ANC in the community (low, high), community poverty (low, high), community education for women (low, high), community media exposure (low, high), and place of living (urban, rural) were included.

### Operational definitions for community level variables

Since the community level components could not be observed or recorded as aggregated data during the survey, all of the components were estimated using their aggregate values from the individual records. Each of them was estimated based on the value of each distinct variable, even if the methodology was the same as in other literatures. A cluster or primary sample unit in the dataset that a group of families shared was referred to in this study as a community level factor. combining components at the individual, group, and community levels to produce variables. Community women's education (percentage of women with primary or post-primary education), community media exposure, community antenatal coverage, and community poverty (percentage of impoverished households) were among the community variables. Other community factors were region and place of residence. The continuous community-level variables were further split into low and high categories using the mean/median value based on their distribution in order to make the results easier to grasp^23–25^.

#### Community women education

The significance of women's educational achievement as a whole is demonstrated by the community's median distribution of educational attainment. It was considered low if the proportion of women in the community with at least a secondary education fell below the median (0–8.33%); it was considered high if it rose beyond the median (8.34–100%).

#### Community media exposure

Individual responses to media exposure via radio or television served as the basis for this media exposure variable. If the proportion of women in the community who were exposed to media was between 0 and 66%, it was considered low; if it was between 6 and 100%, it was considered high.

#### Community ANC utilization rate

Similar to the previous variable, this one is made up of various separate ANC utilization values. If the percentage of women in the neighborhoods who attended at least three ANC visits fell between 0 and 82%, it was considered poor. 0.83 was the median value.

#### Community poverty

The same process is used to derive this variable from each household's wealth index. In a community's two lowest quintiles of wealth, it was deemed high if there were between 56 and 100% of women, and low if there were between 0 and 55% of women.

### Data management and analysis processes

For these crucial phases, we conducted a secondary analysis of the GDHS 2019–2020 using the Kids Records (KR) dataset. Versions 17 and 19 of STATA and Microsoft Excel were used to clean the data. Calculations and descriptive statistics, including frequency and percentages of various variables, were provided via texts, tables, and graphs. Only variables with *p*-values equal to or less than 0.25 were taken into consideration after each independent variable was put through a multilevel mixed effect logistic regression model. The degree of relationship between the dependent and independent variables was assessed using the adjusted odds ratio, and variables were deemed statistically significant if their p-value was less than 0.05.

In the GDHS data, the infant is part of a cluster, and neonates from that cluster shared more characteristics with each other than neonates from other clusters. Thus, the typical regression model's assumptions of observation independence and equal variance across clusters are broken. This suggests that in order to account for between-cluster consequences, a complex model is needed. A multilevel random intercept logistic regression model was created to examine the association between individual-level and community-level variables and the risk that a newborn will not receive postnatal care within two days of birth.

All told, four models were created. Without any explanatory variables, the first model—also referred to as an empty or null model—was fitted. The disparities across communities were lessened as a result of this tactic. To understand community variances, one must understand the null model. We began by determining the extent to which sociocultural factors may be in charge of the observed variations in mothers' MMF practice. Additionally, this model provided as a benchmark for selecting between multi-level and traditional logistic regression and support for the adoption of a multi-level statistical framework.

It was analyzed using the Proportional Change of Variance (PCV), the Log-Likelihood Ratio test (LLR), the Median Odds Ratio (MOR), the Intraclass Correlation Coefficient (ICC), and the AIC. Only individual-level characteristics were included in the second model. Only neighborhood-level elements were present in the third. In contrast, the final (fourth) model included both elements at the individual and community levels. When comparing models using model deviance, the model with the smallest deviation was picked for reporting and result interpretation.

To compare the stacked models, we used deviation (2log likelihood). The intracellular correlation coefficient (ICC) and log-likelihood were used to determine the variance between clusters. The ICC displays the degree of variation among infants who do not get MMF within 24 h. A multilevel binary logistic regression analysis was conducted to identify the individual and societal factors impacting the MMF before 24 h of the interview in infants aged 6–23 months.

Log (πij ÷ 1 − πij) = βo + β1xij + β2xij + …uj + eij, where πij is the likelihood that no MMF will be consumed, and ij is the likelihood that it will. When none of the explanatory factors are present, the influence on MMF is represented by the intercept, or β0. The variables at the individual and community levels for the ith person in group j are βxij, respectively. Also, because the β's are fixed coefficients, a rise in X can result in an increase in the chance of MMF consumption by an additional ß unit. It demonstrates the jth community's random effect—the influence of the community on the mother's decision to intake MMF. Assuming that each community has a unique intercept (β0) and fixed coefficient (β), the clustered nature of the data as well as between and between community variances were taken into consideration.

When the models were compared using the likelihood test, it was discovered that Model 4 had the least DIC value and was the best fit. The final model's mean VIF value, which was used to assess multicollinearity, was 1.53, and all variables had VIF values lower than 10. The crude odds ratio (COR) and adjusted odds ratio (AOR) were used to calculate the relationship between the dependent and independent variables (AOR). For the final model, factors with a COR p-value of less than or equal to 0.25 have been chosen as competitors. The strength of associations between dependent and independent factors was assessed using adjusted odds ratios and 95% confidence intervals with a < 0.05 *p*-value. The median odds ratio (MOR), which is the median value of the odds ratio between the area at the lowest risk and the highest risk when two clusters are randomly chosen, was used to assess the measure of variance. MOR = e0.95√VA or, MOR = exp. [√ (2 × VA) × 0.6745], where; VA is the area level variance^26,27^. The Proportional Change in Variance (PCV reveals the variation in MDD intake among children 6–23 months explained by factors. The PCV is calculated as = $\frac{Vnull-VA}{Vnull}$*100. Where: Vnull is the initial model's variance and VA is the model's variance with additional terms. Also, the Intra Class Correlation Coefficient (ICC), a measurement of the variation in bottle feeding between clusters, is computed as; ICC = VA ÷ VA + 3.29 ∗ 100%, where; VA = area/cluster level variance^26,27^.

### Ethical approval and consent to participate

Online at www.dhsprogram.com, ethical approval and a letter of permission were requested, and the DHS program was given permission through email to access the data for this study. This study made use of freely accessible data that was completely devoid of any personal information. The GDHS provided the secondary data used in the study. Concerns about informed consent, confidentiality, anonymity, and privacy of the study sample were ethically addressed by the GDHS authorities, and we did not alter or utilize the data in any other way. Both participants and the general public were excluded from this investigation. The study's data set was publicly accessible and devoid of any private information. The study is conducted using secondary data from the GDHS. We didn't alter the data and utilized it for other purposes. Both patients and the general public were not involved in this investigation.

## Results

### Sociodemographic characteristics of the study participants

In order to assess the consumption of MMF, a total of 2100 weighted mothers were included in the study. About 683 (32.54%), and 940 (44.77%) of the study's participants, were between the ages of 25 and 29 years old and did not attend formal school respectively. Nevertheless, almost all of them—1969 or 93.79%—were married, and 472 (22.50%) of them were from the poorest households. Furthermore, about 1702 (81.03%) and 1846 (87.93%) participants, respectively, had at least three ANC visits and institutional deliveries, based on the locations where babies are delivered. Around 1733 (82.55%) of study participants reported being employed at the time of the survey, and more than half 1172 (55.86%) of them had reported that their infants had undergone a PNC examination after 2 months. Additionally, 1798 (85.61%) of household heads are male, and 1212 (57.73%) of mothers come from families with ten or more members. Approximately 922 (43.92%), 1094 (52.11%), and 916 (43.64%) of the children in the children's profile were males and were born in the fourth or higher rank, respectively. Between the ages of 11 and 17 months, there are roughly 782 (37.23%) and 2058 (98.02%) infants observed, respectively (Table 1).

**Table 1:** Individual level factors of sociodemographic characteristics of study participants on MMF intake among mothers and 6–23 months old children in The Gambia, GDHS 2019/2020, (n = 2100).

Variables	MMF intake		Total, n (%)
	No, n (%)	Yes, n (%)	
Maternal age in years			
15–19	66 (52.14%)	61 (47.86%)	127 (6.06
20–24	185 (45.04)	226 (54.965)	411 (19.60)
25–29	283 (41.47)	400 (58.53)	683 (32.54)
30–34	175 (39.55)	268 (60.45)	443 (21.09)
35–39	119 (38.39)	192 (61.61)	311 (14.81)
40–44	47 (44.71)	58 (55.29)	104 (4.97)
45–49	7 (34.81)	13 (65.19)	20 (0.93)
Maternal education			
No formal education	786 (83.59)	154 (16.41)	940 (44.77)
Primary	321 (77.11)	95 (22.89)	416 (19.84)
Secondary	512 (77.85)	145 (22.15)	657 (31.30)
Higher	57 (65.85)	29 (34.15)	86 (4.09)
Marital status			
Not married	104 (79.46)	27 (20.54)	131 (6.21)
Married	1571 (79.80)	398 (20.20)	1969 (93.79)
Wealth index			
Poorest	403 (85.33)	69 (14.67)	472 (22.50)
Poorer	368 (83.94)	70 (16.06)	438 (20.88)
Middle	335 (74.87)	112 (25.13)	447 (21.29)
Richer	314 (77.07)	94 (22.93)	408 (19.41)
Richest	255 (76.36)	79 (23.64)	334 (15.92)
Currently working			
No	870 (84.99)	154 (15.01)	1023 (48.73)
Yes	806 (74.84)	271 (25.16)	107 (51.27)
ANC follow up			
Less than four	309 (77.57)	89 (22.43)	398 (18.97)
Four and above	1366 (80.30)	335 (19.70)	1702 (81.03)
Place of delivery			
Home	202 (79.78)	51 (20.22)	253 (12.07)
Health facility	1473 (79.78)	373 (20.22)	1846 (87.93)
Utilization of contraception			
No	1283 (84.72)	231 (15.28)	1515 (72.15)
Yes	497 (84.93)	88 (15.07)	585 (27.85)
Currently breastfeeding status			
No	299 (81.46)	68 (18.54)	367 (17.45)
Yes	1376 (79.43)	357 (20.57)	1733 (82.55)
Postnatal checkup after 02 months			
No	727 (78.45)	200 (21.55)	926 (44.14)
Yes	948 (80.85)	224 (19.15)	1172 (55.86)
Family size			
2–6	318 (81.58)	72 (18.42)	390 (18.54)
7–10	390 (78.19)	109 (21.81)	498 (23.73)
More than 10	968 (79.86)	244 (20.14)	1212 (57.73)
Sex of the household head			
Male	1440 (80.11)	358 (19.89)	1798 (85.61)
Female	235 (77.86)	67 (22.14)	302 (14.39)
Reading to magazine/newspaper			
No	1646 (80.33)	403 (19.67)	2049 (97.61)
Yes	29 (57.46)	22 (42.54)	51 (2.39)
Watching to television			
No	834 (80.36)	204 (19.64)	1038 (49.46)
Yes	841 (79.22)	221 (20.78)	1062 (50.54)
Listing to radio			
No	1035 (80.52)	250 (19.48)	1285 (61.21)
Yes	641 (78.62)	174 (21.38)	815 (38.79)
Child birth weight			
Large	730 (79.16)	192 (20.84)	922 (43.92)
Average	675 (80.53)	163 (19.47)	838 (39.93)
Small	270 (79.63)	69 (20.37)	339 (16.15)
Sex of the child			
Male	867 (79.24)	227 (20.76)	1094 (52.11)
Female	808 (80.37)	197 (19.63)	1006 (47.89)
Birth order			
First	346 (81.01)	81 (18.99)	428 (20.36)
2nd and 3rd	602 (79.59)	154 (20.41)	756 (35.99)
4th and above	727 (79.36)	189 (20.64)	916 (43.64)
Twin status			
No	1636 (79.51)	422 (20.49)	2058 (98.02)
Yes	39 (93.47)	3 (6.53)	42 (1.98)
Number of under five children			
0–2	741 (78.06)	209 (21.94)	950 (45.24)
More than 02	934 (81.21)	216 (18.79)	1150 (54.76)
Child age in months			
6–11	669 (92.20)	57 (7.80)	726 (34.58)
12–17	584 (74.66)	198 (25.34)	782 (37.23)
18–23	422 (71.32)	170 (28.68)	592 (28.20)

### Community level factors characteristics of the study participants

More than half of them, as demonstrated in the following table on community literacy, high media exposure, and low ANC utilization, which is 1366 (65.08%), 1138 (54.17%), and 1127 (53.67%), respectively. Moreover, 866 (41.23%) and 1378 (65.64%) of the study participants, respectively, came from Brikama and urban areas (Table 2).

**Table 2:** Community level factors characteristics of study participants on MMF intake among mothers and 6–23 months old children in The Gambia, GDHS 2019/20, (n = 2100).

Variables	MDD intake		Total, n (%)
	No, n (%)	Yes, n (%)	
Community ANC utilization			
Low	878 (77.89)	249 (22.11)	1127 (53.67)
High	797 (81.98)	176 (18.02)	973 (46.33)
Community poverty			
High	611 (84.89)	109 (15.11)	719 (34.26)
Low	1065 (77.12)	316 (22.88)	1381 (65.74)
Community media exposure			
Low	790 (82.15)	172 (17.85)	962 (45.83)
High	885 (77.78)	253 (22.22)	1138 (54.17)
Community women education			
Low	612 (83.43)	122 (16.57)	734 (34.92)
High	1063 (77.83)	303 (22.17)	1366 (65.08)
Types of places of residence			
Urban	1065 (77.30)	313 (22.70)	1378 (65.64)
Rural	610 (84.52)	112 (15.48)	722 (34.36)
Region			
Banjul	13 (71.35)	5 (28.65)	18 (0.86)
Kanifing	297 (84.26)	55 (15.74)	352 (16.77)
Brikama	639 (73.84)	227 (26.16)	866 (41.23)
Mansakonko	77 (83.33)	15 (16.67)	92 (4.39)
Kerewan	211 (84.67)	38 (15.33)	249 (11.87)
Kuntaur	107 (80.19)	27 (19.81)	134 (6.37)
Janjanbureh	108 (86.58)	17 (13.42)	125 (5.96)
Base	223 (84.60)	41 (15.40)	264 (12.56)

### Random effect analysis of minimal meal frequency

Because the DHS data are hierarchical, we assessed the clustering effect. The null model has a very high ICC in the random-effects study. This shows that variation within clusters only accounts for roughly 12.72% of the diversity in MMF provision for young children, leaving individual variation to explain the remaining 87.28% of the range. The empty model's higher MOR value revealed a wide range in MMF supplies between clusters. A child in the cluster with high MMF utilization had a 1.94 times higher likelihood of using MMF provision than a child in the cluster with lower MMF intake, according to the MOR value of the empty model. If two children from two different clusters were observed, one would be more likely than the other to use MMF provision. The model with the smallest divergence, in this case Model III with a deviation of 2702.72, was found to be the best-fit model. Deviation was also utilized to assess the model's fitness. The decline from 3037.10 to 2702.72 was shown by the model DIC, with an estimated difference of 334.38. The ideal situation necessitates Model III (the final best matched model). (Table 3).

**Table 3:** Individual and community-level factors associated with MMF among 6–23 months old children in The Gambia, GDHS 2019/20, (n = 2100).

Independent variables	Null model	Model I	Model II	Model III
		AOR (95% CI)	AOR (95% CI)	AOR (95% CI)
Maternal age				
15–24		Ref		Ref
25–34		1.03 (0.82, 1.29)		1.05 (0.83, 1.32)
35–49		1.33 (0.99, 1.78)		1.35 (1.01, 1.79) *
Family size^$^		1.00 (0.99, 1.009)		1.00 (0.99, 1.008)
Household head				
Male		Ref		Ref
Female		0.72 (0.53, 0.98)		0.72 (0.53, 0.98) *
Wealth index				
Poorest		Ref		Ref
Poorer		1.08 (0.81, 1.45)		1.13 (0.84, 1.52)
Middle		1.12 (0.81, 1.57)		1.37 (0.96, 1.96)
Richer		1.04 (0.71, 1.53)		1.44 (0.94, 2.22)
Richest		1.09 (0.69, 1.74)		1.76 (1.04, 2.99) *
Educational status				
No formal education		Ref		Ref
Primary		1.44 (1.11, 1.88)		1.44 (1.11, 1.87)*
Secondary, and above		1.46 (1.13, 1.89)		1.43 (1.09, 1.86)*
Contraceptive use				
No		Ref		Ref
Yes		1.18 (0.94, 1.49)		1.14 (0.90, 1.43)
Currently breast feeding				
No		Ref		Ref
Yes		0.01 (0.07, 0.16)		0.10 (0.07, 0.15)*
Currently working status				
No		Ref		Ref
Yes		1.25 (1.02, 1.54)		1.27 (1.04, 1.56)*
Watching to television				
No		Ref		Ref
Yes		1.04 (0.82, 1.32)		1.03 (0.81, 1.31)
Number of under five children				
0–02		Ref		Ref
More than 02		0.84 (0.66, 1.07)		0.79 (0.62, 1.00)
Child size at birth				
Large		0.79 (0.60, 1.04)		0.79 (0.61, 1.04)
Average		1.08 (0.82, 1.42)		1.01 (0.84, 1.45)
Small		Ref		Ref
Child age in month				
6–11		Ref		Ref
12–17		1.44 (1.15, 1.78)		1.40 (1.13, 1.73)*
18–23		1.48 (1.11, 1.96)		1.44 (1.08, 1.91)*
Community women education				
Low			Ref	Ref
High			1.13 (0.85, 1.49)	0.97 (0.71, 1.33)
Community media exposure				
Low			Ref	Ref
High			1.34 (1.04, 1.74)	1.32 (0.99, 1.75)
Regions				
Banjul			Ref	Ref
Kanifing			0.89 (0.51,1.57)	1.02(0.55,1.86)
Brikama			1.25 (0.73,2.13)	1.62(0.91,2.91)
Mansakonko			2.02 (1.11, 3.70)	3.51 (1.77, 6.97)*
Kerewan			2.96 (1.66, 5.30)	5.17 (2.67, 9.99)*
Kuntaur			1.19 (0.66, 2.14)	2.26(1.14,4.47) *
Janjanbureh			1.16 (0.640, 2.09)	2.35 (1.19, 4.64) *
Base			1.16 (0.65, 2.05)	1.79 (0.93, 3.43)
Random parameters and model comparison				
Community-level variance	0.48 (0.31, 0.74)	0.56 (0.36, 0.88)	0.35 (0.21, 0.57)	0.37 (0.22, 0.64)
ICC (%)	12.72	14.73	9.54	10.22
MOR (CI, 95%)	1.94 (1.66, 2.21)	2.05 (1.72, 2.38)	1.75 (1.51, 2.00)	1.79 (1.51, 2.07)
PCV (%)	Reference	− 16.67	27.08	22.92
Log-likelihood (LLR)	− 1518.55	− 1373.87	− 1499.59	− 1351.36
DIC (-2LLR)	3037.10	2747.74	2999.18	2702.72

$, Ref. and * indicates continuous variable, reference variables and significance level at *p*-value < 0.05 respectively.

### Factors associated with MMF among infants and young children in The Gambia

This study found that a number of significant and highly potential determinants, including maternal and child age, wealth index, educational attainment, breastfeeding status, employment status, sex of the household head, and regions, were statistically significant with MMF. It was evident that those mothers who have primary and secondary and higher educational attainment status had revealed higher odds to provide the MMF supply for their infant and young children compared to their counterparts with no formal education enrollment (aOR = 1.44, CI 1.11, 1.87), and (aOR = 1.43, CI 1.09, 1.86) respectively. Similarly, it also revealed that mothers with highest household wealth index and whose age is from 35 to 49 years old have shown higher odds of feeding their infant and young children with more MMF compared to poorest and 15–24 years old mothers by (aOR = 1.76, CI 1.04, 2.99) and (aOR = 1.35, CI 1.01, 1.79) respectively. Conversely, female household heads and currently breast-feeding status have shown a 28%, and 99.9% less likely odds to provide MMF for their infants and young children by the (aOR = 0.72, CI 0.53, 0.98), and (aOR = 0.10, CI 0.07, 0.15) as compared to male household heads and who had not on breast feeding respectively. Furthermore, mothers who are currently worker had shown more positive tendency to provide MMF for their children by the odds of (aOR = 1.27, CI 1.04, 1.56) compared to with mothers who did not have active work status. Children whose age found from 12–17 to 18–23 months old have a higher odd of (aOR = 1.40, CI 1.13, 1.73), and (aOR = 1.44, CI 1.08, 1.91) times to receive the MMF as compared to with 6–11 months old children respectively. The last but not the least associated variable was regions. Participants who are living in Mansakonko, Kerewan, Kuntaur, and Janjanbureh local government areas have shown a higher odd to provide MMF for their infants and young children by (aOR = 3.51, CI 1.77, 6.97), (aOR = 5.17, CI 2.67, 9.99), (aOR = 2.26, CI 1.14, 4.47), and (aOR = 2.35, CI 1.19, 4.64) as compared to Banjul local governmental area respectively (Table 3).

## Discussion

Inappropriate feeding practices increase the risk of undernutrition, sickness, and mortality in babies and young children (6–23 months). The development of substantial measures to avoid undernutrition and the accompanying morbidity and death among these children will benefit from a better understanding of the level of MMF use nationally among children aged 6–23 months in The Gambia. In order to ascertain the prevalence of MMF apply and associated factors among children aged 6–23 months in The Gambia, a countrywide survey was conducted. Last but not least, the current study found that in The Gambia, the prevalence of MMF use among children aged 6–23 months was assessed to be 57.95% (95% CI 55.82, 60.04).

Comparing this result to those from studies conducted in China (75.1%)^28^, Tanzania (82%)^29^, Rwanda (83%)^30^, Pakistan (84.7%)^31^, and a systematic review in Ethiopia (63.8%)^32^, the finding was lower. With the reliable evidence we have on hand, we can back this up. The heterogeneity may result from a variety of factors, including the instruments employed, parental knowledge and attitude, community cultural beliefs towards MMF, sociodemographic features of the study population, and child nutritional management policy. Additionally, the government's and stakeholders' active involvement in the child feeding debate may result in change. This result was higher than those of studies conducted in Nigeria (33.6%)^33^, India (41.5%)^34^, and Burkina Faso (24.37%)^35^, but it was comparable with the study conducted in Ghana (57.3%)^36^.

In The Gambian context, and other similar settings in African countries exploring the minimum meal frequencies and determinants within the broader context of complementary feeding interventions involves considering various factors. Research indicates that inadequate meal frequency is attributed to 20% of children aged 6–11 months and 9% of children aged 12–23 months^37^. Additionally, determinants of inappropriate complementary feeding practices in The Gambia, and other African countries include socioeconomic aspects, household demographics, cultural practices, and caregiver characteristics^38,39^. The occupation of main caregivers, gender roles, and the size of the family have all been linked to complementary feeding habits, particularly when it comes to the number of meals required to meet a child's nutritional needs^39^. In order to address child malnutrition and improve complementary feeding practices, it is imperative to comprehend these determinants^37–39^.

Rich families and working mothers were 1.76 and 1.27 times more likely than poor families to provide children aged 6–23 months the recommended MMF (AOR = 1.76, 95% CI 1.04, 2.99). The research done in Ethiopia and India supported this^32,34^. The concept is distinct and somewhat understandable. The rationale for this action might be that the mother would feed her infant because there is no issue with the food's accessibility. Due of the food's accessibility, even if she is not fully aware of the suggested MMF, there is a potential that she will feed it to her child. Similarly, working mothers are more likely to be economically better off because they have their own income. As a result, it is not difficult for them to eat the recommended MMF for their children compared to no working mothers^40,41^.

Compared to male household heads, female household heads have revealed less likely hood of providing the required MMF for the infants and young children. Similar finding from Ethiopia has been declared^41,42^. Even though the author has faced to cross over with several findings, however, we can support this with solid evidence that we have at hand. If the head of the household is a female, almost all responsibilities of the family will be on her. Therefore, she might spend her time outside or far away from her home to do or work the necessity of the family. Due to these and other implicit factors the probability of spending time outside the home is much and she might not give MMF for her child^43^.

Children between the ages of 12 and 17 and 18 to 23 months had 1.40- and 1.44-times higher odds of receiving the required MMF than those between 6 and 11 months. The study carried out in Bangladesh^44^, Ethiopia^32^, and Malawi^45^ provided evidence in favor of this. This was also corroborated by a study done in Ghana, which found a favorable correlation between a child's age (18–23 months) and attaining the required MMF^46^. This is because many times infants and young children think that it will be difficult for them to eat food and it will cause problems for their health, so they may not start feeding them soon. As they get older, they can leave the mothers breast milk, so the chance of them eating food increases.

Compared to their peers, breastfeeding children were currently 0.99 times less likely to meet the required meal frequency. This may be due to the fact that breastfeeding children's meal frequency is lower than that of non-breastfeeding children. Non-breastfeeding children are required to eat at least four meals per day, whereas breastfeeding children must reduce their meal frequency by at least one meal to meet the requirements. Mothers of breastfed children may believe that they don't require many more items, and they may have recently started using MMF in little doses. On the other side, mothers may have given them a lot of food if a child was not breastfed because of their circumstances.

Additionally, in line with a study done in Malawi^47^, and Ethiopia^48^, children whose mothers were older had a higher likelihood of achieving the MMF and minimal dietary diversity. Compared to younger women, older mothers are more likely to have experience and expertise on how to raise their children. Therefore, such strong links between maternal age and supplemental feeding practices imply that the mother's experience may be a major factor in improper baby and early child feeding practices. An additional study conducted among Indian population validated this conclusion^49^.

This study found that mothers who have attended their primary and secondary or higher educational attainment have shown higher chance to provide the recommended MMF for their children as compared to mothers without formal educational enrollment. This finding supported by studies done in Malawi, Ghana, and Ethiopia^41,43,47,50^. Educated mothers or parents are more open to learning new things, are more aware of the value of good child-feeding habits, and can modify their conduct more quickly than illiterate ones who are more static and take longer to do so.

The MMF of infants and young children aged 6–23 months was similarly related to local government districts. Compared to mothers who had lived in Banjul, mothers who had lived in Mansakonko, Kerewan, Kuntaur, and Janjanbureh, were 3.51, 5.17, 2.26 and 2.35 times more likely to provide the required MMF for their children the necessary variety of meals. The author was unable to locate any prior material to support this finding because there has never been a nationwide study of MMF in The Gambia. However, the demographics of these local government areas provide us with ample justification. The 2018 Multiple Indicator Cluster Survey (MICS) report has shown that although young children from Banjul had slightly higher exclusive breast-feeding rate, however, regarding MMF, percent currently breastfeeding and receiving solid, semi-solid or soft foods, and percent appropriately breastfed according to age, the local government area of Banjul has revealed a lower performance than the remaining regions^8^. In contrast, facts show that there are more mothers working for the government formally or informally in the Banjul regions than in other regions, and that these mothers are less likely to give their children the recommended MMF despite a varied diet. Studies suggested that mums who worked for the government would provide their children a more varied diet but less frequently. This is a result of working mothers spending a lot of time away from their children at work^29,43^. To incorporate underserved communities, The Gambian government updated its national nutrition policy for the years 2010 to 2020. "A The Gambia free of malnutrition" is the policy's stated goal. The strategy to improve the nutritional and health status of children implements a number of key strategies to end hunger, food insecurity, and malnutrition, including: promoting the use of nourishing, safe, and locally accessible complementary foods, raising public awareness of the significance of optimal infant and young child feeding, advocating for the creation of an environment that facilitates optimal infant and young child feeding^8,51,52^.

In order to improve awareness of healthy eating habits and care practices for parents, care givers, and healthcare professionals, care, implementation and policy revision are extended outside of medical institutions and into the homes and communities where these mothers and their infants reside, with a focus on both case prevention and treatment. A continuation of UNICEF's assistance to the Government of The Gambia on maternal and infant nutrition as well as the management of severe acute malnutrition (SAM), the Post-Crisis Response to Nutrition Insecurity Project is supported by the EU. The impact of this policy's implementation was significant, particularly for rural mothers^8,53^.

### Strength, and limitations of the study

The hierarchical form of the data and the fact that the data are nationally representative give this study its strength. We employed the necessary advanced statistical analysis by considering both individual, and community level factors. The research participant sample is also appropriate. The study has some drawbacks, including recall bias caused by participant self-report and a one-day, 24-h recall that failed to capture the child’s regular eating patterns. The recall bias, and, being a self-reported investigation all together, it might not provide exact figures for MMF practices in The Gambia by affecting the women’s accurate past feeding experience to their children. Although, DHS uses a trained data collector professionals, interviewer, and social desirability biases might have a chance of affect the results. Furthermore, the use of secondary data limited us to incorporate other important explanatory variables such cultural, and contextual related. it might not accurately reflect participants’ past feeding habits.

## Conclusion

In conclusion, this study has concluded: the implementation of MMF in The Gambia among infants and young children aged 6–23 months is inadequate compared to WHO recommendations and national goals. Our results in The Gambia make it very evident that many children under the age of two and breast feeders are not receiving the MMF, one of the key indications of complementary feeding practices. These results, however, indicate that the majority of the variables included in this study contributed to a more accurate prediction of MMFs. Future research should therefore focus more on other factors related to the country's regional structures, cultural contexts, and demography. The components of MMF practices were often connected with the age of the child, mother's age, education, occupation, home affluence, mother's working position, breast-feeding mother, sex of the household head, and region. It is strongly advised to improve child complementary feeding practices through the use of micronutrient supplements, food security, healthcare use, and improved socioeconomic status. Similar initiatives that support economically underprivileged homes and mothers who are illiterate would have a positive impact on the MMF initiative. Policymakers will be more successful in their efforts if they can identify additional determinants.

## Data Availability

Data for this analysis came from the DHS Program on The Gambian 2019/20 DHS data files, which are open to the public. The DHS program repository has the datasets created and/or analyzed during the current investigation at www.dhsprogram.com.
